# Supplementary material for: Cohort profile: The Dutch wound monitor cohort and the Swedish Region Halland Integrated Platform (RHIP) wound cohort
Source: PLoS One. 2026 Jan 21;21(1):e0339260. doi: 10.1371/journal.pone.0339260 (PMC12822990; doi:10.1371/journal.pone.0339260)
Supplement: Attachment — Submitted filename: Response to Reviewers.pdf [file pone.0339260.s001.pdf]

# Response to Reviewer Comments

Authors of the Manuscript

We thank the reviewers for their thoughtful and constructive feedback. Below, we address each comment in detail and describe the corresponding changes made to the manuscript.

## Academic Editor

**Academic editor Comment:** Please ensure that your manuscript meets PLOS ONE's style requirements, including those for file naming.

**Author Response:** Thank you for this comment. We have gone through the style requirements and made sure that we meet them.

**Academic editor Comment:** Thank you for stating the following financial disclosure:

"This work was conducted as part of the CAISR Health Research Profile at Halmstad University with funding made possible by the Knowledge Foundation (grant number 20200208 01H)."

Please state what role the funders took in the study. If the funders had no role, please state: "The funders had no role in study design, data collection and analysis, decision to publish, or preparation of the manuscript."

If this statement is not correct you must amend it as needed.

Please include this amended Role of Funder statement in your cover letter; we will change the online submission form on your behalf.

**Author Response:** Thank you for your comment, we have included an amended Role of Funder statement in the cover letter. Thank you also for changing the online submission form

**Academic editor Comment:**

Thank you for stating the following in the Acknowledgments Section of your manuscript:

"This work was conducted as part of the CAISR Health Research Profile at Halmstad University with funding made possible by the Knowledge Foundation (grant number 20200208 01H)."

We note that you have provided funding information that is currently declared in your Funding Statement. However, funding information should not appear in the Acknowledgments section or other areas of your manuscript. We will only publish funding information present in the Funding Statement section of the online submission form.

Please remove any funding-related text from the manuscript and let us know how you would like to update your Funding Statement. Currently, your Funding Statement reads as follows: "This work was conducted as part of the CAISR Health Research Profile at Halmstad University with funding made possible by the Knowledge Foundation (grant number 20200208 01H)." Please include your amended statements within your cover letter; we will change the online submission form on your behalf.

**Author Response:** Thank you for your feedback. We have now removed the funding text from the manuscript and and updated the cover letter with an amended funding statement.

**Academic editor Comment:** Please review your reference list to ensure that it is complete and correct. If you have cited papers that have been retracted, please include the rationale for doing so in the manuscript text, or remove these references and replace them with relevant current

references. Any changes to the reference list should be mentioned in the rebuttal letter that accompanies your revised manuscript. If you need to cite a retracted article, indicate the article's retracted status in the References list and also include a citation and full reference for the retraction notice.

**Author Response:** We have added three references in the new manuscript. It is nr 6,7 and 8. All of them are referenced in the new paragraph in the Introduction section where we situate the the cohorts within the broader landscape of cohorts as per reviewer 1s comment.

## Reviewer 1

**Reviewer Comment:** The article is positioned as a "cohort profile," yet also discusses potential AI modelling applications. While this is valuable, the primary purpose (descriptive vs. preparatory for modelling) should be explicitly clarified in the Introduction and Conclusion. Consider please, stating more clearly how these datasets are expected to contribute to improving wound care workflows or informing policy.

**Author Response:** Thank you for pointing this out. It should have been more clearly stated that the aim is to characterize the two wound cohorts while the reflections around AI-modeling is about a promising avenue for utilizing the cohorts that is only meant to be a discussion point. In the revised version we have rewritten the abstract, introduction and conclusion to make this more clear. These changes can be found in the last seven rows of the abstract, the last three rows of the third paragraph in the introduction and the first six rows of the conclusion.

**Reviewer Comment:** Regarding Ethics and Data Access, and we noticed that ethical approvals are listed, but we consider that the manuscript should explicitly address data privacy measures, especially given the use of real-world clinical data and potential re-identification risks in smaller subgroups. - just a reflection

**Author Response:** We appreciate this reflection. In the revised version of the manuscript there is now a paragraph about measures taken to ensure data-privacy. Parts of it were moved from inclusion and exclusion criteria section and other parts were added after considering your reflection. The paragraph is in the last paragraph of the ethical statement section.

**Reviewer Comment:** Please clarify or explain the rationale for comparing Dutch and Swedish data (beyond availability) should be explained, particularly regarding differences in healthcare delivery and wound care practices. Methodological Detail and Transparency

**Author Response:** Thank you for this comment. Although this was not sufficiently clear in the article, it is not immediately relevant for the authors that they come from Sweden and the Netherlands. The rationale for presenting these two wound cohorts in conjunction is that they provide two complementary perspectives of the patient and wound care journey. In the revised version we attempt to explain this more clearly, the explanation of the rationale can be found in the method section in the first sentence of the second paragraph.

**Reviewer Comment:** We fekt the need to have a better description or more detail on data cleaning, quality control, and missing data handling is warranted. For example, how were errors or inconsistencies in wound classification addressed? Please, clarify whether any harmonization of variable definitions was attempted between cohorts for comparative purposes.

**Author Response:** This is a good point. We have now clarified our approach which, with regards to quality control, data cleaning, missing data and harmonization. It can be found in the

discussion section under the first new subsection. We decided not to put it in the method section because of the need to reference figures and tables which is not allowed to do until just before presenting them ackording to PLOS ONEs standards. Additionally, parts of rationale for certain decisions are more of a discussion-like nature.

**Reviewer Comment:** Contextualization and Literature Integration should be improved since i fekt the need to better situate these cohorts within the broader landscape of wound databases internationally. Are there similar registries (e.g., U.S., U.K., Australia) and how do these compare in scope or structure?

**Author Response:** Thank you for this comment. We agree that we should describe what we know about other registries. In the revised version there is now description of what we know about other cohorts. It can be found in the last paragraph of the Introduction section. Note that in this paragraph we have added three new references to the reference list

**Reviewer Comment:** Last suggestuoins are associated with Limitations and Biases, because while some dataset limitations are acknowledged, additional discussion of selection bias (e.g., home-care patients in the Dutch cohort vs. broader care settings in RHIP) would contextualize findings.

**Author Response:** This is a fair point and should have been adressed in the original manuscript. The other reviewer wanted some more reflection on the generalizability of the potential findings. We added a few sentences about that in the dicussion and also nuanced it with the selection bias issue mentioned here. It can be found in the discussion section, in the last seven rows under the headline "Information driven care and cross-cohort analysis".

## Reviewer 2

**Reviewer Comment:** Enhance the clarity of Figure 3 – the contrast between RHIP and Wound Monitor could be more intuitive to general readers.

**Author Response:** Thank you for this comment, we agree that it was not clearly demonstrated what the main contrasts are for general readers. We have edited the image with the aim to make it more clear what differences and similarites there are between the databases. The image is still called image 3 in the revised manuscript.

**Reviewer Comment:** Include a concise summary table comparing the wound classification systems used in both cohorts (e.g., ICD-10 vs. NPIAP/UT)

**Author Response:** Thank you for this suggestion. We have discussed it and decided that we believe it is better to keep the presentation as it is with one classification tree for each database. This is because there is no clear overlap between the systems for the subcateogries of wound types. However the comment is well taken and we have added an explanation for this decision in the manuscript. The explanation can be found in the first paragraph under the Wound Classification section.

**Reviewer Comment:** Briefly address the generalizability of findings outside Sweden and the Netherlands, especially in regions with limited digital infrastructure.

**Author Response:** Thank you for this comment. We agree that this is an important point. In the revised version of the manuscript there is now a reflection on the generalizability of findings across other parts of the world. It can be found in the discussion section, in the last seven rows under the headline "Information driven care and cross-cohort analysis".

**Reviewer Comment:** Clarify how future NLP/LLM efforts might resolve missing wound ID and outcome variables in RHIP.

**Author Response:** Thank you for your comment. We agree that this was not clarified enough in the article. In the revised version of the manuscript we have added a few sentences describing why we expect this to be possible and how we plan to deal with the inherent uncertainty in such an effort. This reflection can be found in the third to last paragraph under the content, quality, quantity and granularity headline. It is the sentence starting with "Discussion with clinicians have indicated..." and ends at the end of that paragraph.

We hope that these revisions adequately address the reviewers' concerns and improve the clarity and rigor of our manuscript. We are grateful for the opportunity to revise and resubmit.

Sincerely,
The Authors
